# Delayed Cancer Registration and Estimation of Screening Colonoscopy Effects

**DOI:** 10.1001/jamanetworkopen.2024.35669

**Published:** 2024-10-01

**Authors:** Hermann Brenner, Thomas Heisser, Michael Hoffmeister

**Affiliations:** 1 Division of Clinical Epidemiology and Aging Research, German Cancer Research Center, Heidelberg, Germany; 2 German Cancer Consortium, German Cancer Research Center, Heidelberg, Germany

## Abstract

This cohort study assesses the magnitude of cancer registration delays and their estimated affects on the NordICC trial results assessing the long-term effects of screening colonoscopy in preventing colorectal cancer.

## Introduction

Epidemiological studies have long suggested strong effects of screening colonoscopy in preventing colorectal cancer (CRC).^1,2^ However, much weaker than expected effects were reported in 2022 from the NordICC trial, the first and so far only randomized clinical trial reporting on long-term effects of screening colonoscopy, to our knowledge.^3^ In the NordICC trial, CRC was ascertained by record linkage with cancer registries. Registration delays are a well-known common concern in population-based cancer registration.^4^ We aimed to explore the magnitude of registration delays and their estimated impact on reported effects from the NordICC trial.

## Methods

Details on the design, data collection, ethics review, informed consent, and analysis of the NordICC trial have been reported elsewhere.^3^ Briefly, 85 179 men and women aged 55 to 64 years without a previous diagnosis of CRC, as assessed by national registries, were identified in 3 Northern European countries (Poland, Norway, and Sweden) in 2009 to 2014 and randomly assigned in a 1:2 ratio to receive or not receive an invitation to undergo a single screening colonoscopy. Among these, there were 221 postrandomization exclusions due to a prior CRC diagnosis identified after randomization.

We estimated the magnitude of cancer registration delay at baseline by comparing numbers of postrandomization exclusions of individuals with CRC diagnoses prior to randomization with expected CRC diagnoses per year (derived by multiplying age-specific incidence rates from cancer registries with numbers of trial participants).^5,6^ We furthermore derived the expected outcomes associated with comparable delay during follow-up in the observed 10-year CRC risk difference and numbers needed to invite to colonoscopy or to undergo colonoscopy to prevent 1 CRC event. Calculations were conducted in January 2024 using Microsoft Excel 2013.

## Results

Approximately 80 new CRC diagnoses would have been expected within 1 year in the NordICC study population, based on cancer registry data in the years of recruitment (Table 1). Post hoc exclusion of almost 3 times as many individuals with CRC (221 individuals) who had not been identified by record linkage with the cancer registries at the time of randomization suggests a 2- to 3-year delay of cancer registration.

**Table 1.** Calculation of Expected Number of Incident CRC Events per Year at Baseline in the NordICC Trial Population^a^

Metric	Poland	Norway	Sweden	Total
CRC incidence, No. per 100 000 population per y				
Age 55-59 y	63.5	94.2	63.9	NA
Age 60-64 y	106.1	143.8	102.2	NA
Participants, No.				
Age 55-59 y	28 792	12 524	1784	43 100
Age 60-64 y	25 736	13 887	1862	41 485
Expected CRC events/y^b^				
Age 55-59 y	18.3	11.8	1.1	31.2
Age 60-64 y	27.3	20.0	1.9	49.2
Age 55-64 y	45.6	31.8	3.0	80.4

Abbreviations: CRC, colorectal cancer; NA, not applicable.

^a^ Cancer registry data extracted from ECIS database for Poland^5^ and from NORDCAN database for Norway and Sweden^6^; ECIS data for Poland were available for calendar years 2009 to 2013, and NORDCAN data for Norway and Sweden were available for calendar years 2009 to 2014; all incidence data pertain to both sexes combined.

^b^ Expected numbers of cases calculated as (incidence × participants) / 100 000.

Table 2 shows reported results after 10 years of follow-up and results that would have been expected accounting for potential delay in cancer registration by 1, 2, or 3 years. CRC risk differences of 0.22% in intention-to-screen and 0.38% in per-protocol analyses were reported, suggesting that 455 people would be needed to be invited and 263 people would need to undergo colonoscopy to prevent 1 CRC event. The reported risk differences exclusively emerged in the 4-year period from 6 to 10 years of follow-up. A mean registration delay by 1, 2 to 3 years would imply that only approximately 75%, 50%, or 25% of individuals diagnosed in that 4-year period would have been registered, that the true risks and the emerging risk difference within that period would have been underestimated by approximately 25%, 50%, or 75%, and that true numbers needed to invite for colonoscopy or to undergo colonoscopy would be approximately 25%, 50% or 75% lower than reported numbers.

**Table 2.** Estimation of 10-Year Colorectal Cancer Risk Reduction and Numbers Needed to Invite to Screening Colonoscopy and to Undergo Colonoscopy to Prevent 1 Colorectal Cancer Event, Assuming Various Degrees of Mean Delay in Cancer Registration

Metric	Assumed mean delay in cancer registration			
	None^a^	1 year	2 y	3 y
Intention-to-screen analysis				
10-y risk difference, %	0.22 (0.07-0.37)	0.29	0.44	0.88
No. needed to invite	455 (270-1429)	341	227	114
Adjusted per-protocol analysis				
10-y risk difference, %	0.38 (0.20-0.58)	0.51	0.76	1.52
No. needed to scope	263 (172-500)	197	132	66

^a^ Results (95% CI) reported in the NordICC trial.^3^

## Discussion

The findings of this study suggest that delay of cancer registration most likely led to major underestimation of 10-year CRC risk reduction in the analyses of the NordICC trial reported in 2022, in which ascertainment of incident cases had most likely remained incomplete for a large proportion of the participants recruited in 2009 to 2014. Limitations of our analyses include lack of possibility of exact quantification of and correction for registration delay. Updated analyses ensuring complete 10- and 15-year follow-up will be crucial to derive the true reductions of CRC risk and mortality in the trial’s predefined interim and primary analysis. In the meantime, available estimates are to be interpreted with caution, as they likely severely underestimate true screening colonoscopy effects.

## Notes

Supplement.
Data Sharing Statement
