# Supplementary material for: Delayed Cancer Registration and Estimation of Screening Colonoscopy Effects
Source: JAMA Netw Open. 2024 Oct 1;7(10):e2435669. doi: 10.1001/jamanetworkopen.2024.35669 (PMC11445683; doi:10.1001/jamanetworkopen.2024.35669)
Supplement: Supplement. — Data Sharing Statement [file jamanetwopen-e2435669-s001.pdf]

Data Sharing Statement

Brenner. Underestimation of Screening Colonoscopy Effects Due to Delayed Cancer Registration. *JAMA Netw Open*. Published October 01, 2024. doi:10.1001/jamanetworkopen.2024.35669

Data
Data available: No

Additional Information
Explanation for why data not available: All data used for our analyses are published or publicly available from the cited references.
